# Biotechnological approaches for artemisinin production in *Artemisia*

**DOI:** 10.1007/s11274-018-2432-9

**Published:** 2018-03-27

**Authors:** Waqas Khan Kayani, Bushra Hafeez Kiani, Erum Dilshad, Bushra Mirza

**Affiliations:** 1 Department of Plant Breeding, Swedish University of Agricultural Sciences, Växtskyddsvägen 1, 230 53 Alnarp, Sweden; 2 Department of Bioinformatics and Biotechnology, International Islamic University, Islamabad, 45320 Pakistan; 3 Department of Biosciences, Capital University of Science and Technology (CUST), Islamabad, Pakistan; 4 Department of Biochemistry, Faculty of Biological Sciences, Quaid-i-Azam University, Islamabad, 45320 Pakistan

**Keywords:** Artemisinin, *Artemisia*, Hairy roots, Cell cultures, Transgenics, Biotechnology

## Abstract

**Abstract:**

Artemisinin and its analogues are naturally occurring most effective antimalarial secondary metabolites. These compounds also possess activity against various types of cancer cells, schistosomiasis, and some viral diseases. Artemisinin and its derivatives (A&D) are found in very low amounts in the only natural source i.e. *Artemisia* plant. To meet the global needs, plant sources have been exploited for the enhanced production of these natural products because their chemical synthesis is not profitable. The generally adopted approaches include non-transgenic (tissue and cell cultures) and transgenic together with the cell, tissue, and whole transgenic plant cultures. The genes targeted for the overproduction of A&D include the biosynthetic pathway genes, trichome development genes and *rol* genes, etc. Artemisinin is naturally produced in trichomes of leaves. At the same time, transgenic hairy roots are considered a good source to harvest artemisinin. However, the absence of trichomes in hairy roots suggests that artemisinin biosynthesis is not limited to trichomes. Moreover, the expression of the gene involved in trichome development and sesquiterpenoid biosynthesis (TFAR1) in transgenic and non-transgenic roots provokes researchers to look for new insight of artemisinin biosynthesis. Here we discuss and review precisely the various biotechnological approaches for the enhanced biosynthesis of A&D.

**Graphical Abstract:**

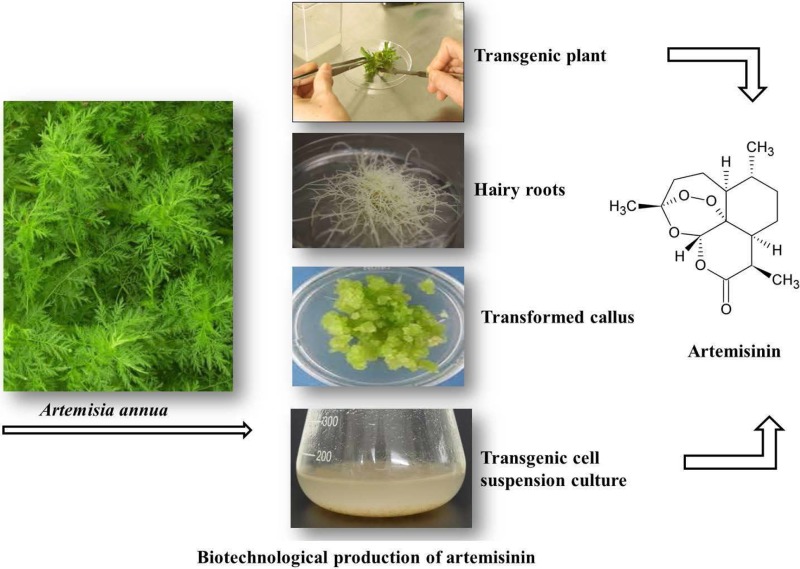

## Introduction

Artemisinin is an antimalarial pro-drug which has been considered the last line of defence against malaria for many decades (Bryant et al. 2015). Whereas, the other older generation antimalarial drugs are now found less effective due to the acquired resistance against them (Fidock 2010). Artemisinin, a sesquiterpene lactone is the core component of artemisinin-based combination therapies (ACTs) especially for the treatment of multidrug-resistant malaria (Eckstein-Ludwig et al. 2003). With the passage of time, there is a significant rise in the demand for ACTs, as can be estimated from the fact that the number of ACT treatments rose to 36 fold during 2005 to 2013 in endemic countries, reaching a total of 392 million in 2013 (WHO 2017). But the reality seems to be far more challenging when it comes to the reliable supply of artemisinin as the precursor compound for the active ingredient of ACTs. This fact is no doubt of crucial importance in the fight against malaria, which is the fifth most prevalent disease in underdeveloped countries and tenth overall cause of death. This scenario is projected to stay at the same level till 2030 (Nahlen et al. 2005). According to WHO, 216 million cases of malaria occurred globally in 2016 and it led to 445,000 deaths (WHO 2017).

Artemisinin is naturally found in the aerial parts of the plant, i.e. flowers, leaves, stems, buds and seeds (Ferreira et al. 1995a) in amounts ranging from 0.1 to 0.8% of the dry weight of the plant (Abdin et al. 2003), maximally reaching 1.5% in some cases (Kumar et al. 2004; Weathers and Towler 2012). Ontogeny and phenology play important role in the production of secondary metabolites in plants. Its synthesis is basically carried out in the glandular trichomes (GLTs), present on flowers, floral buds, and leaves and sequestered in the subcuticular sac at the apex of the GLTs. Trichome-specific fatty acyl-CoA reductase 1 (TAFR1) is thought to be involved in GLT development and sesquiterpenoid biosynthesis; both are important for artemisinin production (Ferreira et al. 1995a; Olsson et al. 2009).

**Fig. 1:**
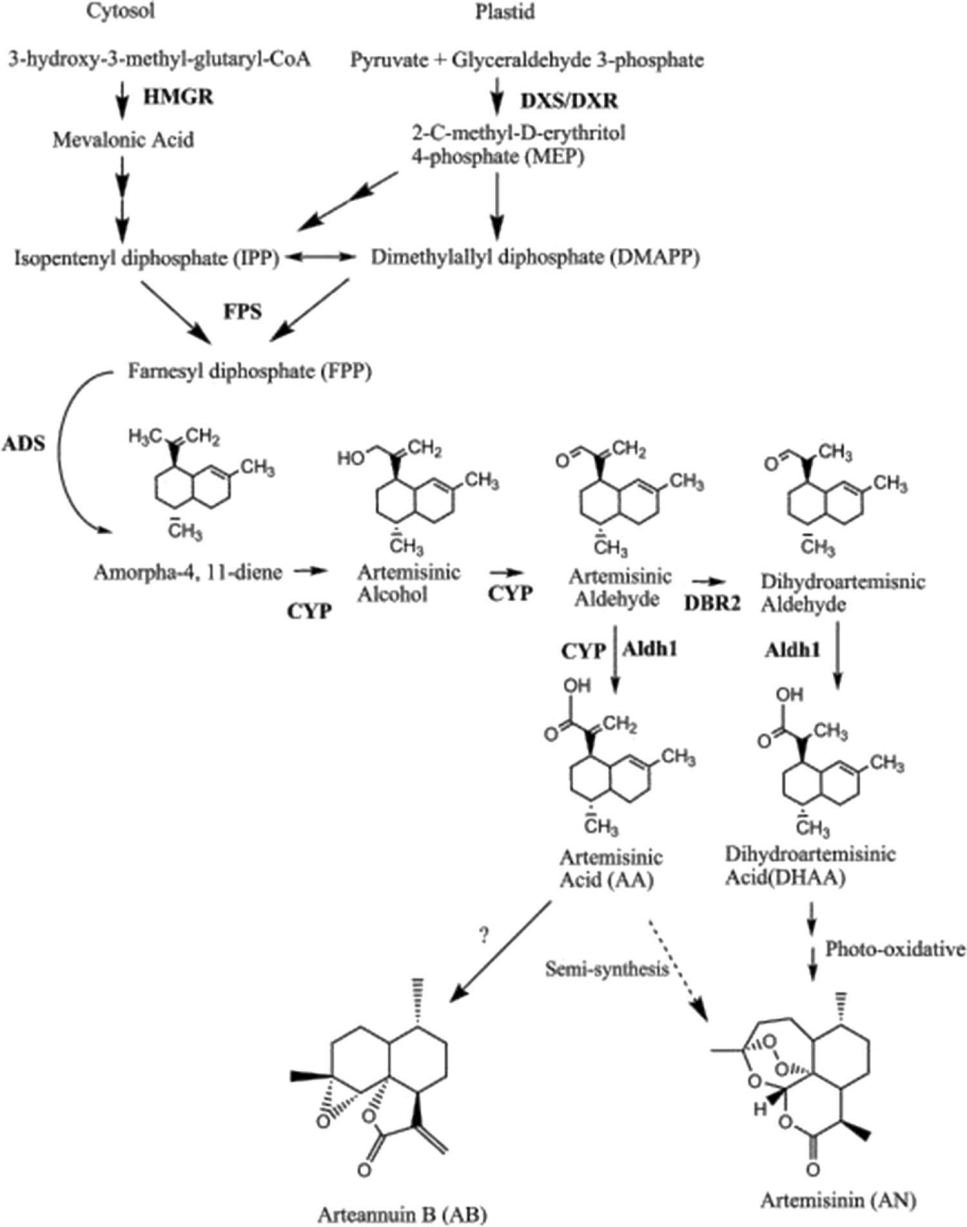
A schematic diagram showing isopentenyl diphosphate and artemisinin biosynthetic pathway (Arsenault et al. 2010a)

Artemisinin biosynthesis is almost completely known (Nguyen et al. 2011) (Fig. 1). It originates from a common biosynthetic precursor for the synthesis of terpenoids that is isopentenyl diphosphate (IPP), formed via either the cytosolic mevalonate pathway (MVA) or the plastidic mevalonate-independent pathway (MEP); both pathways supply the IPP for artemisinin (Croteau et al. 2000). Condensation of three molecules of IPP to farnesyl diphosphate (FDP) is catalyzed by farnesyl diphosphate synthase (FPS). Amorphadiene synthase (ADS) a sesquiterpene cyclase then catalyzes the formation of amorpha-4,11-diene by the cyclization of FDP. The next two steps are catalyzed by a cytochrome P450, CYP71AV1 (CYP). Amorpha-4,11-diene is oxidized to the artemisinic aldehyde and also to artemisinic acid (AA) (Teoh et al. 2006), which is then converted by a double-bond reductase (DBR2) to dihydroartemisinic aldehyde, the precursor to dihydroartemisinic acid (DHAA) (Zhang et al. 2008). Conversion of DHAA to AN is a non-enzymatic photo-oxidative reaction involving reactive oxygen species (ROS), which by adding three oxygen atoms leads to the formation of the endoperoxide pharmacophore of AN (Brown and Sy 2007; Wallaart et al. 2001).

The production of artemisinin is compromised because particularly in third world countries the only reliant source of its production is *Artemisia* plant with several limitations. First of all, being a competitor against food for the use of land it will cause a rise in food prices thus giving less incentive to farmers for its cultivation. Additionally, the plan for *A. annua* cultivation has to be designed somehow 14 months before the drugs can be produced. In this regard, natural disasters like flood may be the limiting factor making the artemisinin production unpredictable (Noorden 2010). As far as the chemical synthesis of artemisinin is concerned that has been found to be complex and expensive and thus have not been supplanted as the favored method. Although the chemical synthesis of artemisinin is reported (Zhu and Cook 2012), to date the plant remains the only source of this drug. On the account of all these factors, different setups are being developed for the inexpensive production of artemisinin, which is not reliant on the cultivation of *A. annua* and should have the capability to get scaled up when required (Table 1).

**Table 1:** Review articles describing biosynthesis of A&D and their uses

S. No.	Titles	References
01	Sesquiterpene lactones from *Artemisia* genus: biological activities and methods of analysis	Ivanescu et al. (2015)
02	Secondary metabolism of hairy root cultures in bioreactors	Kim et al. (2002)
03	Artemisinin: current state and perspectives for biotechnological production of an antimalarial drug	Liu et al. (2006)
04	Medicinal Importance of *Artemisia absinthium* Linn (Afsanteen) in Unani Medicine: A Review	Ahmad et al. (2010)
05	Metabolic engineering of artemisinin biosynthesis in *Artemisia annua* L.	Liu et al. (2011)
06	The molecular mechanism of action of artemisinin-the debate continues	O’Neill et al. (2010)
07	The *Artemisia* L. Genus: a review of bioactive essential oils	Abad et al. (2012)
08	*Artemisia dracunculus* L. (Tarragon): a critical review of its traditional use, chemical composition, pharmacology, and safety	Obolskiy et al. (2011)
09	Dried-leaf *Artemisia annua*: A practical malaria therapeutic for developing countries?	Weathers et al. (2014)
10	Artemisinin production in *Artemisia annua*: studies in planta and results of a novel delivery method for treating malaria and other neglected diseases	Weathers et al. (2011)
11	Recent advances in artemisinin production through heterologous expression	Arsenault et al. (2008)
12	Trichomes + roots + ROS = artemisinin: regulating artemisinin biosynthesis in *Artemisia annua* L	Nguyen et al. (2011)
13	Transgenic approach to increase artemisinin content in *Artemisia annua* L.	Tang et al. (2014)
14	Secondary metabolites of *Artemisia annua* and their biological activity	Bhakuni et al. (2001)
15	Artemisinin, a novel antimalarial drug: biochemical and molecular approaches for enhanced production	Abdin et al. (2003)
16	Artemisinin production in *Artemisia annua*: studies in planta and results of a novel delivery method for treating malaria and other neglected diseases	Weathers et al. (2011)
17	Artemisinin: the biosynthetic pathway and its regulation in *Artemisia annua*, a terpenoid-rich species	Weathers et al. (2006)
19	Use of whole plant *Artemisia annua* L. as an antimalarial therapy	Mostafa et al. (2012)
20	Oxidative stress in malaria and artemisinin combination therapy: Pros and Cons	Kavishe et al. (2017)
21	A review of biotechnological artemisinin production in plants	Ikram and Simonsen (2017)
22	Malaria and artemisinin derivatives: an updated review	Tayyab Ansari et al. (2013)
23	New insights into artemisinin regulation	Lv et al. (2017)
24	Anticancer Activity of Artemisinin and its Derivatives	Slezakova and Ruda-Kucerova (2017)
25	*Plasmodium falciparum* Resistance to Artemisinin Derivatives and Piperaquine: A Major Challenge for Malaria Elimination in Cambodia	Valentine et al. (2016)
26	Antimalarial qinghaosu/artemisinin: The therapy worthy of a Nobel Prize	Krungkrai and Krungkrai (2016)

So far, three different approaches have been practiced for increased production of A&D, including non-transgenic, transgenic and heterologous transgenic systems (Arsenault et al. 2008). Non-transgenic approaches include the selective breeding of high artemisinin-containing elite varieties of *A. annua*, manipulation of growth condition, use of in vitro cultures, and elicitation to use plant’s natural defence system. In transgenic approach, transgenics of different *Artemisia* species with a variety of genes have been produced. While, in the heterologous systems, key genes of artemisinin biosynthetic pathway are inserted into organisms other than *A. annua*. Here we discuss the so far progress made on the biotechnological approaches (transgenic and heterologous transgenic systems) for enhanced artemisinin production.

## Non-transgenic approaches

Highly exploited medicinal compounds, artemisinin, and their derivatives are vastly studied for their antimalarial and anticancer effects. So far, precursor molecules of a pathway, intermediate compounds of a pathway or chemicals like methyl jasmonate (MeJ), chitosan and salicylic acid (SA) have been used in a majority of the elicitation studies to enhance a number of secondary metabolites. Non-transgenic approaches are considered easy to practice with low-level ethical constraints. Wild aerial part, in vitro raised plants and greenhouse acclimatized plants of *A. amygdalina* revealed the presence of artemisinin (Rasool et al. 2013). But, wild inflorescences and calli did not show the presence of artemisinin. Lualon et al. (2008) regenerated untransformed plants of *A. annua* using 0.1 mg/l thidiazuron (TDZ) and found artemisinin content of 3.36 µg/mg dry weight which is two-fold higher than that of in vitro grown plants of the same age. However, when *ex vitro* grown untransformed plants of *A. annua* were elicited with chitosan oligosaccharide (COS) and salicylic acid (SA), COS up-regulated the transcriptional levels of the genes ADS and TTG1 2.5 fold and 1.8 fold after 48 h individually, whereas SA only up-regulated ADS 2.0 fold after 48 h (Yin et al. 2012). Additionally, the supplementation of dimethyl sulfoxide (DMSO) to the untransformed seedlings increased artemisinin in the shoots of *A. annua* (Mannan et al. 2010b). Rooting of untransformed *A. annua* shoots (SAM clone) by the supplementation of a-naphthaleneacetic acid increases trichome size on leaves and helps drive the final steps of the biosynthesis of artemisinin (Nguyen et al. 2013).

Baldi and Dixit (2008) reported that elicitation of cell cultures of *A. annua* (untransformed) with mevalonic acid (MA) (50 mg/L) resulted maximally an increase of twofold of artemisinin content in comparison to control, while a maximum increase of 3.47 fold in artemisinin was attained when MeJ (5 mg/L) was added. Combined supplementation of MA and MeJ resulted in maximum artemisinin production of 96.8 mg/L which was 4.79 times higher than control callus cultures (20.2 mg/L). However, some researchers suggest that elicitation might not be a good method to enhance artemisinin because it triggered biosynthesis after a long time (48 h). Another study conducted on *A. annua* suggests roots (which neither produce significant artemisinin nor its precursor compounds) to regulate artemisinin production in the leaves. Researchers grafted roots of lines with high artemisinin-producing leaves to the low artemisinin producing shoots and observed an increased leaf production of artemisinin in those low artemisinin-producing plants (Wang et al. 2016). A wide range of various reports describing the A&D production and its uses is given below.

## Transgenic approaches

### Transformation with genes involved in artemisinin biosynthetic pathway (ABP)

#### Transgenic *Artemisia*

Modifications have been made in plants by improving the expression of endogenous pathways or by introducing novel genes to modify its pathways (Ikram and Simonsen 2017). Researchers from numerous groups have almost resolved the difficult task of biosynthesis of artemisinin. Now, noticeable progress has been observed in the regulation of biosynthesis of artemisinin and underlying mechanisms (Bouwmeester et al. 1999). Key enzymes and their genes which are main precursors and are necessary for biosynthesis of artemisinin i.e. [farnesyl diphosphate synthase (FPS), amorpha-4,11-diene synthase (AMS)], and the genes of the enzymes linked with the molecular basis of biosynthesis of artemisinin including squalene synthase (SQS), have been cloned from *A. annua* (Matsushita et al. 1996; Mercke et al. 2000; Wallaart et al. 2001; Yan et al. 2003). Dhingra and Narasu (2001) isolated the main enzyme used in the biochemical conversion of arteannuin B to artemisinin. The genes of artemisinin biosynthesis including ADS, CYP71AV1, DBR2, and ALDH1 are all preferably articulated in the glandular trichomes (Covello et al. 2007; Olsson et al. 2009).

Several groups have shown that transformation with artemisinin biosynthetic pathway genes can increase artemisinin production through modification of its biosynthetic pathway (Ikram and Simonsen 2017). Overexpression of FPS in *A. annua* leads to the accumulation of the increased amount of artemisinin through conversion of IPP and DMADP into FDP (Maes et al. 2011). Biosynthesis of artemisinin can be improved significantly by an enhanced expression of both HMGR and ADS instantaneously through a co-transformation experiment in *A. annua* (Alam and Abdin 2011); furthermore, production of artemisinin can also be improved by overexpressing both HMGR and FPS together in *A. annua* (Wang et al. 2011). In the MEP pathway, DXR has been considered as an important rate-limiting enzyme effectively used to increase the production of monoterpenoids such as peppermint essential oils (Hasunuma et al. 2008). Genetic map of *A. annua* further proved that DXR was strongly interconnected with the production of artemisinin (Graham et al. 2010). Chen et al. (1999) raised hairy roots of *A. annua* plants after the infection of engineered *A. rhizogenes* containing *fds* gene. The transgenic hairy roots showed an overexpression of FPS and an improved production of artemisinin content i.e. 3 to fourfold higher than control. Further, Chen et al. (2000) reported 2 to threefold increase in artemisinin production in *A. annua* plants transformed with same FPS gene through *A. tumefaciens* mediated genetic transformation method.

The cloning and characterization of *AaHDR* involved in the increased production of artemisinin through enhanced production of artemisinin biosynthesis precursors via MEP pathway in *A. annua* has been reported (Peng et al. 2011). It will be helpful to understand more about the function of HDR at the level of molecular genetics and unveil the biosynthetic mechanism of artemisinin. Genetic engineering can be used for enhanced artemisinin production through an increase in expression of the key genes of the enzymes used for the artemisinin biosynthesis, such as FPS and AMS, in the transgenic high-yield *A. annua*.

**Fig. 2:**
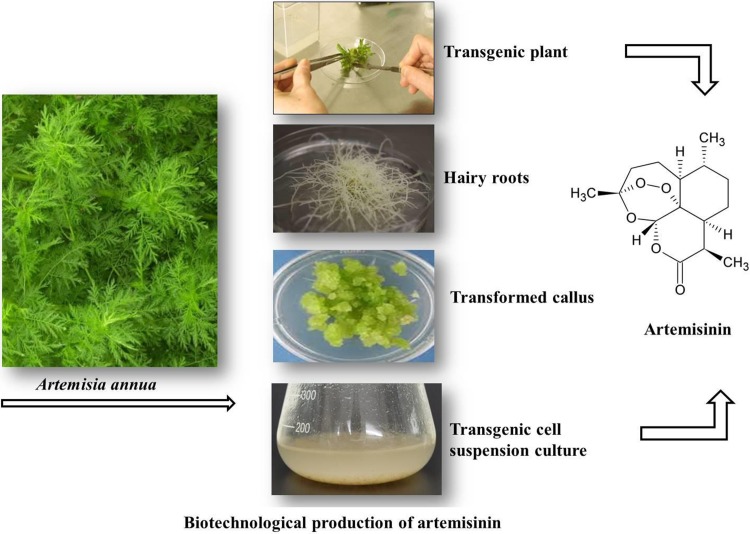
Transgenic approaches in the biotechnological production of artemisinin

As chemical synthesis of A&D is not economically feasible (Delabays et al. 2001), wild plants contain scarce amount of these secondary metabolites (Mannan et al. 2010a) and ~ 40% of the global population is threatened by malaria (Dhingra et al. 1999), there is a high demand for these compounds in the international market. To satisfy the vast need for medicine, various transgenic biotechnological approaches are discussed here (Fig. 2).

#### Heterologous systems

Progressive achievements in molecular biology enabled scientists to explore new expression systems for artemisinin biosynthesis beyond its only source on the planet earth i.e. *A. annua*. Genetic engineering of the molecular pathway(s) leading to the synthesis of artemisinin has been attempted in several organisms including plants, yeast, and bacteria to improve its production. Arsenault et al. (2008) has explained in detail different approaches using heterologous expression systems for the enhanced production of artemisinin and focused on newer methodologies of genetic engineering of artemisinin biosynthetic pathway genes to meet the demands for the treatment of different diseases especially malaria.

Heterologous expression system especially *Escherichia coli* and *Saccharomyces cerevisiae* have been studied so far for this purpose (Arsenault et al. 2008). It is a promising technology for the production of important compounds; however, monomeric proteins can be easily produced in microbes while the production of complex secondary metabolites needs the reconstruction of the metabolic pathway. Various approaches have been developed to express the sesquiterpene lactones in microbial systems including fungi (Asadollahi et al. 2008) and bacteria (Picaud et al. 2007). However, there are few reports describing the cloning of genes into microbial hosts specifically for the biosynthesis of artemisinin and its derivatives. Majority of these reports reveal the expression of amorpha-4,11-diene which is a volatile precursor of artemisinin. In a report, a 9-genes biosynthetic pathway of amorpha-4,11-diene was introduced in *E. coli*. Keeping in mind the loss of gaseous phase, microbes were cultured in a two-phase partitioning bioreactor and 89% pure amorpha-4,11-diene was procured in ~ 0.5 g/L concentration of the culture medium (Newman et al. 2006). A semi-synthetic approach of artemisinin by using strains of *S. cerevisiae* for biological production of artemisinic acid and its chemical conversion to artemisinin is also reported (Paddon et al. 2013). Ro et al. (2006) engineered *S. cerevisiae* mevalonate pathway with amorphadiene synthase (*ADS*) and cytochrome P450 monooxygenase (*CYP71AV1*) to produce artemisinic acid. They found higher levels of artemisinic acid (up to 100 mg/L) in reconstructed yeast than *A. annua*. Further, a cDNA clone was generated from the mRNA of trichomes encoding cytochrome P450 (*CYP71AV1*) and expressed in *S. cerevisiae* which catalyzed the oxidation of various intermediates of artemisinin biosynthetic pathway including amorpha-4,11-diene (Teoh et al. 2006). Although there are some successful reports for the production of various secondary metabolites in reconstructed microbes, the expression of complex pathway limits the heterologous expressions of the non-native molecule (Newman et al. 2006).

### Transformation with genes not involved in artemisinin biosynthetic pathway (ABP)

An efficient methodology to genetically target the desired plant metabolites is the metabolic engineering, which is also a hot spot for genetic engineering of different medicinal plants. Different attempts have been taken to increase artemisinin production in *Artemisia* species through the insertion of different genes affecting flowering (Wang et al. 2007), phytohormone levels (Sa et al. 2001) and farnesyl diphosphate synthesis (Chen et al. 2000).

#### Transformation with flowering genes

*Artemisia* species were transformed with different flowering stimulating genes because it has been established that artemisinin production is higher during the flowering season. Wang et al. (2007) has transformed *A. annua* with an early flowering gene CONSTANS (CO) and the flowering promoting factor 1 gene (*fpf1*), both from *Arabidopsis*, using *A. tumefaciens*. Though *fpf1* transformed plants showed flowering 20 days earlier than non-transgenic plants, yet no major difference in artemisinin production was observed. *CO* transformed plants also showed the similar results with early flowering in transformed plants but no significant increase in artemisinin concentration depicting direct relation between flowering and production of artemisinin.

#### Transformation with genes of phytohormones

Genes encoding phytohormone or phytohormone were also found triggering enhanced production of A&D. Transformation of *A. annua* plants through *A. tumefaciens* containing the Isopentenyl transferase gene (*ipt*) resulted in 70% increase of artemisinin concentration than control with an increase in chlorophyll content as well (Sa et al. 2001). Likewise, Singh et al. (2016) studied the effect of expression of β-glucosidase gene (*bgl*1) in *A. annua* and found that *bgl*1 induced trichome density (up to 20% in leaves and 66% in flowers) and artemisinin content (up to 1.4% in leaf and 2.56%/g dry weight in flowers) of transgenic plants than the control plants.

#### Transformation with the genes of trichome development

**Fig. 3:**
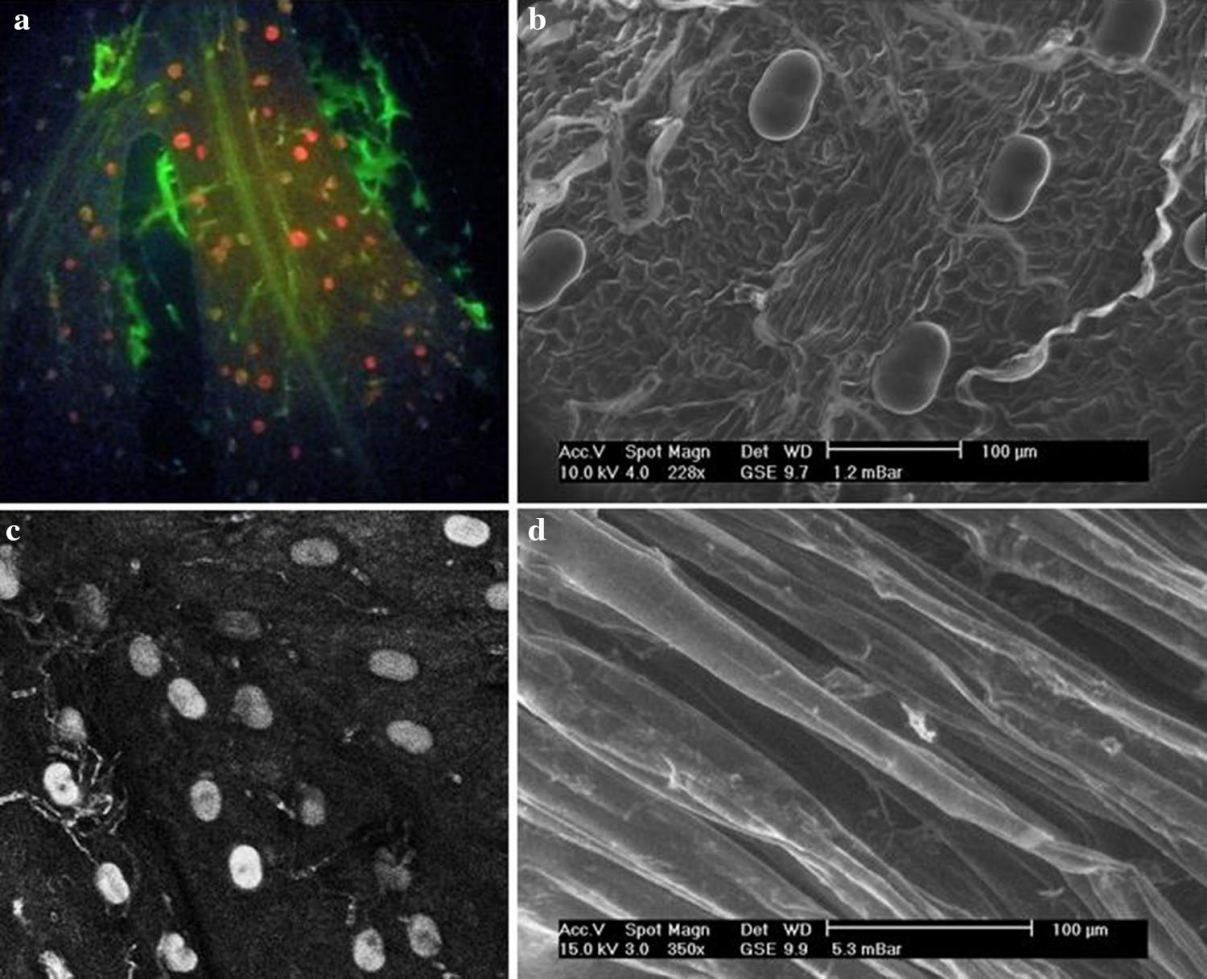
Trichomes present on leaf surfaces of *Artemisia* species (**a**–**c**), while transgenic hairy roots are devoid of trichomes (**d**). **a** Fluorescent microscopy of leaf of transgenic *A. annua* containing *rol* genes, **b** environmental scanning electron microscopy of the leaf of transgenic *A. annua* harboring *rol* genes, **c** confocal microscopy of leaf of transgenic *A. annua* containing *rol* genes, **d** environmental scanning election microscopy of leaf of untransformed in vitro grown *A. annua*

Artemisinin is produced in glandular trichomes, which are about 10-cells in size located on leaves, floral buds, and flowers (Ferreira et al. 1995b; Olsson et al. 2009; Tan et al. 2015; Tellez et al. 1999) and sequestered in the epicuticular sac which is present at the apex of the trichome (Olsson et al. 2009). Presence of trichomes in aerial parts of *Artemisia* species and their absence in roots supports the higher accumulation of artemisinin in the aerial parts of *Artemisia* species (Fig. 3). For example, the leaves which are younger and later in growth level have the higher amount of artemisinin as compared to the mature leaves formed during the early stages of plant’s development; this difference was accounted for higher trichome density and a higher capacity per trichome in the upper leaves (Lommen et al. 2006). Trichome-specific fatty acyl-CoA reductase 1 (TFAR1) is known to stimulate trichome development and to catalyze sesquiterpenoid biosynthesis (Maes et al. 2011). Dilshad et al. (2015b) supported the idea of overexpression of TFAR1 genes and hence artemisinin production. Another report by Dilshad et al. (2015a) explained the effect of a higher expression of TFAR1 gene in *A. carvifolia* is directly linked to the enhanced production of artemisinin. There are several other reports on overexpression of trichome genes and enhanced production of artemisinin. Tan et al. (2015) transformed *A. annua* with *TRICHOME AND ARTEMISININ REGULATOR 1* (*TAR1*) gene and observed a significant increase in artemisinin production and also suggested that this gene is directly linked to artemisinin pathway genes at some stages of development. Besides TFAR1 and *TAR1* transformations, Liu et al. (2009) reported that amount of glandular trichomes can be improved by overexpressing putative TTG1 gene related to significant increase in artemisinin production in *A. annua* leaves. Overexpression of an important trichome-specific transcription factor (AaORA) in *A. annua* both glandular secretory trichomes and nonglandular T-shaped trichomes (TSTs) is highly expressed in transformed lines and acts as a positive regulator in the biosynthesis of artemisinin (Lu et al. 2013).

### *rol* genes

#### Hairy roots raised through the infection of *Agrobacterium rhizogenes*

Hairy roots syndrome appears after the infection of *Agrobacterium rhizogenes*, which induces the plagiotropic roots having increased growth rate and multiple ramifications. Transgenic hairy roots are somewhat similar in morphology to the normal untransformed roots and they have been raised for the commercial-scale production of secondary metabolites (Sharma et al. 2013). In early studies, Weathers et al. (1994) obtained several transgenic root cultures of *A. annua* from the infection of *A. rhizogenes* strain ATCC 15834 and found the biosynthesis of artemisinin up to 0.42% of dry weight (DW). They also detected artemistene, artemisinic acid and arteannuin B and suggested that the commercial production of these compounds using transformed roots is feasible. A rapidly growing clone (YUT16) of these hairy roots (diploid) was used to produce four stable tetraploid clones of *A. annua*. These tetraploid clones revealed a better growth rate and produced up to six times more artemisinin than the diploid parent suggesting the importance of ploidy level in artemisinin biosynthesis (De Jesus-Gonzalez and Weathers 2003). Further, the diploid clones of YUT16 were grown in nutrient mist reactors and resulted in an increase of ~ 3 times as much artemisinin (2.64 µg/g DW) as roots grown in bubble column reactors (0.98 µg/g DW) (Kim et al. 2001). When cultures of this hairy root line were grown in continuous light, they showed a substantial increase in deoxy-d-xylulose-5-phosphate synthase (DXPR) transcript levels compared to dark-grown cultures suggesting plastid-localized artemisinin biosynthetic pathway for its higher biosynthesis (Souret et al. 2002). Growth kinetics study of these roots (Weathers et al. 1994) showed that the use of gibberellic acid (GA3) at 0.01 mg/L (~ 28.9 µM) increased the growth rate of hairy roots of *A. annua* by 24.9% (Smith et al. 1997). This genetically stable roots clone (YUT16) reveals that there are many factors like the ploidy level, reactors type, position of the roots, presence or absence of light and elicitors which effect artemisinin production.

Hairy roots of in vitro generated *A. annua* plants were also obtained by Ahlawat et al. (2012) with the infection of *A. rhizogenes* strain LBA-9402 and revealed maximum artemisinin volumetric biosynthesis of 390 µg/L/d. In another report, transformed root cultures of *A. annua* established by the infection of *A. rhizogenes* strain LBA 9402 revealed production of artemisinin up to 0.004% dry weight (DW). However, supplementation of GA3 in medium provoked artemisinin biosynthesis up to 400% of the control value (Paniego and Giulietti 1996).

Besides artemisinin, its other derivatives have also been studied in transgenic hairy root cultures of *Artemisia* species. Banerjee et al. (1997) infected the leaves of *A. annua* with *A. rhizogenes* strain LBA 9402 and the leaf developed hairy roots which have the ability to produce artemisinic acid and arteannuin B. Interestingly, they found that regenerants of transgenic hairy roots represented a higher growth rate and produced more of these secondary metabolites than the respective mother hairy root clone. In another attempt, transgenic hairy roots of *A. annua* were obtained from NCIB 8196 or MAFF 03-01724 strain of *A. rhizogenes*. Among them, some of the clones were grown in dark, produced undetectable level while some clones cultured in light and in liquid medium produced signals of artemisinin in GC-MS analysis (Jazir et al. 1995). Similarly, Shaneeja et al. (2014) has reported an improved amount of artemisinin in hairy roots of *A. annua* obtained after the infection of two different *A. rhizogenes* species.

Being the intrinsic property of *Artemisia* species to produce artemisinin, other species of *Artemisia* have also been exploited to harvest artemisinin. Mannan et al. (2008) raised transgenic hairy roots by infecting the plants of *A. dubia* and *A. indica* with *A. rhizogenes* strains LBA 9402 and 8196. Hairy roots induction was found higher in infected with LBA9402 as compared to 8196. However, roots of *A. dubia* infected either with LBA9402 or 8196 produced maximum artemisinin (0.603% and 0.753% of DW, respectively). Contrary to it, relatively lower amount of artemisinin was harvested in in vitro regeneration of *A. indica* hairy roots obtained by the infection of strain 8196 in the liquid medium. Those in vitro raised hairy roots resulted in highest root fresh weight as well as artemisinin content (3.9 g and 0.042%, respectively). However, transgenic hairy roots obtained from *A. dubia* explants with the infection of *A. rhizogenes* strain 9402 revealed a higher growth rate (twofold) and a higher production of artemisinin content (36.581 µg/g DW) as compared to control untransformed roots which did not show any artemisinin content (Kiani et al. 2012).

#### Elicitation of the hairy roots

Elicitors or signal molecules are usually the chemicals that modify cell metabolism in order to enhance the biosynthesis of secondary metabolites in a plant cell or tissue cultures. They enable plants in better adaptation to the stress conditions. Certain elicitors have been employed to the cell or hairy root culture of *Artemisia* species for enhanced production of artemisinin and its derivatives. Weathers et al. (1997) studied the effect of four different factors to optimize conditions to harvest maximum root biomass and terpenoid production in transgenic A. annua hairy root clones (YUT16). They found that 15 mM nitrate, 1.0 mM phosphate, 5% sucrose content (wt/vol) and 8 days old cultured roots are the best conditions to harvest maximum biomass as well as terpenoid content.

Besides the above-optimized factors, other elicitors/signal molecules have been used to raise the A&D amount in transgenic hairy roots of *Artemisia* species. Hairy roots induced by the leaf disc method of *A. annua* showed an enhanced production of artemisinin (550 mg/L) when elicited with a homogenate of *Aspergillus oryzae* (Liu et al. 1997). In another report, diploid clones of highly exploited YUT16 hairy roots obtained from *A. annua* (Weathers et al. 1994) when subjected to measure the effect of a broad range of phytohormones on growth of *A. annua* hairy roots and their artemisinin content which revealed that GA3 (0.029 µM) produced the highest values of growth while 2-isopentenyladenine triggered artemisinin biosynthesis more than twice that of the B5 controls, and more than any other hormone studied (Weathers et al. 2005). Hairy roots of *A. annua* obtained from the seeds of YU strain represented sustained and rapid growth when conditioned medium was fed to the roots and the presence of 1% CO_2_ in the carrier gas did not enhance the growth kinetics but it did prevent necrosis of the tissue at the highest mist cycle (Wang and Weathers 2007). GA3 also affected the A&D production in other species of *Artemisia*. Hairy root culture of *A. dubia* obtained by the infection of *A. rhizogenes* strain LBA9402 represented highest artemisinin accumulation of 80 ± 3 µg/g of DW (91% increase) when cultured on media containing GA3 (0.001 mg/L) while 79 ± 3 µg/g of DW was found at 0.138 mg/L of salicylic acid separately as compared to control (Ali et al. 2012).

Supplementation of growth medium with different carbon sources was also found affecting A&D production. It is found that glucose-stimulated artemisinin production maximally in diploid and tetraploid clones of YUT16 hairy root strain while growth in sucrose and fructose was significantly better than in glucose (Weathers et al. 2004). *A. annua* seedlings obtained from the seeds of YU strain produced a 200% increase in artemisinin content by glucose as a carbon source in the medium as compared to sucrose and suggested that these sugars also act as signals to affect the downstream production of artemisinin (Wang and Weathers 2007). The biosynthesis of artemisinin is increased in response to exogenous glucose supplementation in *A. annua* seedlings obtained from the seeds of YU cultivar of *A. annua* by upregulating FPS, DXS, DXR, ADS and CYP transcript levels (Arsenault et al. 2010b). Weathers et al. (2012) found that monosaccharides i.e. glucose increased artemisinin biosynthesis in both the seedlings and hairy root cultures of *A. annua* plants by increasing the expression of some of the genes in the artemisinin biosynthetic pathway. However, disaccharides were found inhibiting the artemisinin biosynthesis which suggests that monosaccharides play a dual role; not only fulfill the carbon requirement for plants but also as a signal switching on signal transduction of artemisinin biosynthetic pathway.

#### Hairy roots in different bioreactors

Different bioreactors have been used to grow plant cells (transformed and untransformed) and this technology has been adopted on a wide scale for the production of A&D. The hairy root cultures obtained from leaf discs (Liu et al. 1997) of *A. annua* were cultivated in a flask, a bubble column, a modified bubble column and a modified inner-loop airlift bioreactor and artemisinin contents in the latter two culture conditions were found higher among the four i.e. 536 mg/L after 20 days (Liu et al. 1998). Highly studied hairy root clone YUT16 obtained by Weathers et al. (1994) was grown at 1 L in disposable culture bag mist reactor showed growth rates higher than that of shake flasks (Sivakumar et al. 2010). Although expression in reactors was equivalent to or greater than that of root cultures of YUT16 grown in shake flasks, surprisingly, transcriptional regulation of HMGR, DXS, DXR, and FPS was greatly affected by the position of the roots in each reactor (Souret et al. 2003).

### *rol* genes carrying transgenic plants

Over the past decade, *rolA, rolB* and *rolC* genes have shown to possess the property of overproduction of secondary metabolites in transformed plant cells. Plants growth and metabolism appeared to be associated with the *rolA* protein, which acts as a stimulator of secondary metabolism (Altvorst et al. 1992; Schmülling et al. 1993). The *rolB* protein plays an important role in the pathway of signal transduction of auxin due to its tyrosine phosphatase activity (Filippini et al. 1996). Estruch et al. (1991) described that *rolC* has cytokinin glucosidase activity and it can be associated with the release of active cytokinins from their inactive glucosides. *RolC* has also been shown to be involved in the production of tropane alkaloids (Bonhomme et al. 2000), pyridine alkaloids (Palazón et al. 1998b), indole alkaloids (Palazón et al. 1998a), ginsenosides (Bulgakov 2008) and anthraquinones (Bulgakov et al. 2002, 2003; Shkryl et al. 2008) in transformed plants and plant cell cultures. Regarding the *rolD* gene, its main function is the conversion of ornithine to proline due to its ornithine cyclodeaminase enzyme activity (Trovato et al. 2001).

Numerous reports have shown that hairy roots cultures can produce a significant amount of secondary metabolites in different plant species transformed with *Agrobacterium rol* genes (Giri and Narasu 2000; Oksman-Caldentey and Sévon 2002). There are few reports about the transformation of *Artemisia* with *rol* genes for production of artemisinin. Our group is working in this direction for several years, and we are successfully producing artemisinin through *rol* genes transformation of different *Artemisia* species using *A. tumefaciens* and *A. rhizogenes*.

Using *Agrobacterium tumefaciens* mediated genetic transformation technology, *A. carvifolia* was transformed with GV3101 strain harboring *rolB* and *rolC* genes for enhanced production of artemisinin and its derivatives. Artemisinin content was increased from 3 to sevenfold in transgenics transformed with the *rol B* gene, and 2.3 to sixfold in those transformed with the *rolC* gene (Dilshad et al. 2015a). We have also shown a 2 to ninefold increase in artemisinin content of *A. annua* plants transformed with *rolB* genes and about fourfold increase in artemisinin amount in *rolC* genes transformed plants (Dilshad et al. 2015b). Besides this, an increase in artemisinin content of *A. dubia* transformed with *rolABC* genes using *A. tumefaciens* has been reported (Kiani et al. 2012). The *rol* genes were found to overexpress intermediate pathway genes of artemisinin biosynthetic pathway. This idea can be supported by Kiani et al. (2016) who transformed *Artemisia annua* and *A. dubia* with *rolABC* genes and the leaves of transgenic plants revealed an increase in artemisinin content up to ninefold when compared to untransformed plants. Interestingly, transgenic plants expressed *CYP71AV1* and *ALDH1* levels higher than that of *ADS*. Moreover, the level of the *TFAR1* expression and trichome density was also significantly increased in all transgenic lines.

We transformed different *Artemisia* plants with *rol* genes which are responsible for enhancing production of secondary metabolites in plants. The exact mechanisms for the action of the *rol* genes are not yet clear (Bulgakov 2008) but it has been suggested that they act through the stimulation of the plant’s defense response which includes induction of many of the hormonal pathways. This may explain why the transformation with *Agrobacterium rol* genes has such a significant effect on the amount of artemisinin produced by the transformed plants. Stimulation of the synthesis of artemisinin within these plants has also allowed us to alter our perception of how and where artemisinin is produced within the plants. This report might help to develop better strategies to increase the production of this valuable therapeutic drug, which will, in turn, allow greater use of it in the chemotherapy of malaria and other diseases.

## Conclusions and perspectives

Artemisinin, nowadays, seems to be the only reliable treatment for multidrug-resistant malaria. Millions of deaths of malarial patients demand a reliable supply of artemisinin, which is intrinsically biosynthesized in *Artemisia* species especially in the trichomes present on the leaves. Due to the limitations of the chemical synthesis of artemisinin, different approaches have been practiced for enhanced production of artemisinin including generation of high artemisinin-containing elite varieties of *A. annua*, manipulation of growth condition for the plant tissues, use of in vitro cultures and elicitation to use plant’s natural defence system. However, various transgenic approaches were also developed to enhance artemisinin production including generation of hairy root cultures. Hairy roots revealed better growth rate and artemisinin production and unveiled many factors affecting A&D production like the ploidy level, reactors type, position of the roots, presence or absence of light, different strains of *A. rhizogenes* and elicitors. Elicitors have also been used to increase the artemisinin biosynthesis either alone or in combinations. Various elicitors have been tried to enhance A&D biosynthesis including GA3, COS, DMSO, nitrates, phosphates, sucrose, TDZ, MeJ, chitosan, SA, and MA and found maximally increasing ~ twofold of artemisinin by the transcriptional upregulation of ADS and TTG1. Interestingly, glucose rather than sucrose was found not only fulfilling the carbon requirement but also driving the signal transduction of artemisinin biosynthetic pathway via upregulation of FPS, DXS, DXR, ADS and CYP genes. Transgenic roots clones, either elicited or unelicited, differed in A&D production in the different bioreactors e.g. flask, a bubble column, a modified bubble column and a modified inner-loop airlift bioreactor, nutrient mist reactors and bubble column reactors. The difference in bioreactors also affected transcriptional regulation of HMGR, DXS, DXR, and FPS differently.

*Artemisia* species have been transformed with a variety of artemisinin biosynthetic pathway genes (FPS, HMGR, ADS, *FDS*, AMS, SQS, and *AaHDR*), trichomes development genes (TTG1, AaORA and *TAR1*) and their overexpression were found increasing A&D production. Majority of these genes either help in conversion of IPP and DMADP into FDP or regulate MEP pathway. However, ADS, CYP71AV1, DBR2, and ALDH1 are preferably expressed in the glandular trichomes. Various *Artemisia* clones were transformed with the genes other than artemisinin biosynthetic pathway including early flowering gene CO, *Ipt* and *fpf1*. However, early flowering did not increase artemisinin concentration but *ipt* resulted in an increase in 70% of artemisinin concentration. *rol* genes, on the other hand, holds the property of overproduction of secondary metabolites in transformed cells. They are playing role in signal transduction of auxin and reported for the enhancement of a vast variety of secondary metabolites. There are few reports regarding the transformation of *Artemisia* with *rol* genes for enhanced production of artemisinin. Our group is working in this direction for several years and we are successfully producing artemisinin through *rol* genes transformation of different *Artemisia* species using *A. tumefaciens* and *A. rhizogenes. rol* genes induced an overexpression of TFAR1 in *A. annua* and *A. carvifolia* and resulted in enhanced production of artemisinin. *rolB* gene is reported as the most powerful inducer of secondary metabolism and artemisinin content was found increased up to 7–9 fold in *rolB* transgenics as compared to *rolC* transgenic plants. Interestingly, transgenic plants expressed higher transcript levels of *CYP71AV1, ADS*, and *ALDH1* which can be compared to higher *TFAR1* expression and trichome density. However, heterologous expression systems for A&D biosynthesis have been studied in *E. coli* and *S. cerevisiae*. Majority of these reports reveals the expression of amorpha-4,11-diene, a volatile precursor of artemisinin. In another semi-synthetic approach, *S. cerevisiae* is used for the production of artemisinic acid and its chemical conversion to artemisinin. Expression of complex pathway declines the heterologous expressions of the non-native molecule. Stimulation of the synthesis of artemisinin within *Artemisia* plants has also allowed us to alter our perception of how and where artemisinin is produced within the plants. This report might help to develop better strategies to increase the production of this valuable therapeutic drug, which will, in turn, allow greater use of it in the chemotherapy of malaria and other diseases.

## Abbreviations

*DXR*: 1-Deoxy-d-xylulose 5-phosphate reductoisomerase
*DXS*: 1-Deoxy-d-xylulose 5-phosphate synthase
ALDH1: Aldehyde dehydrogenase 1
AMS: Amorpha-4,11-diene synthase
ADS: Amorphadiene synthase
*AaHDR*: *Artemisia annua* 1-hydroxy-2-methyl-2-(E)-butenyl 4-diphosphate reductase
AA: Artemisinic acid
A&D: Artemisinin and its derivatives
ACTs: Artemisinin based combination therapies
AN production: Artemisinin production
COS: Chitosan oligosaccharide
CO: CONSTANS
CYP: Cytochrome
DXPR: Deoxy-d-xylulose-5-phosphate synthase
DHAA: Dihydroartemisinic acid
DMSO: Dimethyl sulfoxide
DMADP: Dimethylallyl diphosphate
DBR2: Double bond reductase-2
DW: Dry weight
FPS: Farnesyl diphosphate synthase
FDP: Farnesyl diphosphate
*fpf1*: Flowering promoting factor 1 gene
GA3: Gibberellic acid
GSTs: Glandular secretory trichomes
GLTs: Glandular trichomes
HMGR: Hydroxymethyl glutaryl coenzyme A reductase
IPP: Isopentenyl diphosphate
*ipt*: Isopentenyl transferase gene
MeJ: Methyl jasmonate
MEP: Mevalonate independent pathway
MVA: Mevalonate pathway
MA: Mevalonic acid
TSTs: Non-glandular T-shaped trichomes
ROS: Reactive oxygen species
*rol* genes: Root locus genes
SA: Salicylic acid
SQS: Squalene synthase
TDZ: Thidiazuron
TAR1: TRICHOME AND ARTEMISININ REGULATOR 1
TFAR1: Trichome-specific fatty acyl-CoA reductase 1
WHO: World Health Organization
